# Supplementary material for: Early-Onset Versus Late-Onset Preeclampsia in Bogotá, Colombia: Differential Risk Factor Identification and Evaluation Using Traditional Statistics and Machine Learning
Source: Biomedicines. 2025 Aug 12;13(8):1958. doi: 10.3390/biomedicines13081958 (PMC12383967; doi:10.3390/biomedicines13081958)
Supplement: Supplementary file 1 [file biomedicines-13-01958-s001.zip › biomedicines-3701325-supplementary.pdf]

Supplementary Materials

Early-Onset Versus Late-Onset Preeclampsia in Bogotá, Colombia: Differential Risk Factor Identification and Evaluation Using Traditional Statistics and Machine Learning

Ayala-Ramírez Paola [1,2,*,†], Mennickent Daniela [3,4,*,†], Farkas Carlos [3], Guzmán-Gutiérrez Enrique [2,4,5], Retamal-Fredes Eduardo [6], Segura-Guzmán Nancy [7], Roca Diego [8], Venegas Manuel [7], Carrillo-Muñoz Matias [7], Gutierrez-Monsalve Yanitza [7], Sanabria Doris [9], Ospina Catalina [10], Silva Jaime [10], Olaya-C. Mercedes [11] and García-Robles Reggie [1,2,12]

[1] Human Genetics Institute, Faculty of Medicine, Pontificia Universidad Javeriana, Bogotá 110231, Colombia
[2] Red Iberoamericana de Alteraciones Vasculares en Trastornos del Embarazo (RIVATREM), Chillán 3780000, Chile
[3] Departamento de Ciencias Básicas y Morfología, Facultad de Medicina, Universidad Católica de la Santísima Concepción, Concepción 4090541, Chile
[4] Grupo Inicial de Investigación en Tecnología e Innovación en Salud para el Bienestar de las Personas (VITALIS), Universidad Católica de la Santísima Concepción, Concepción 4090541, Chile
[5] Departamento de Bioquímica Clínica e Inmunología, Facultad de Farmacia, Universidad de Concepción, Concepción 4070386, Chile
[6] Tecnología Médica con Mención en Imagenología y Física Médica, Facultad de Medicina, Universidad Católica de la Santísima Concepción. Concepción 4090541, Chile
[7] Magíster en Ciencias Biomédicas, Facultad de Medicina, Universidad Católica de la Santísima Concepción, Concepción 4090541, Chile
[8] Nutrición y Dietética, Facultad de Medicina, Universidad Católica de la Santísima Concepción, Concepción 4090541, Chile
[9] Research Seedbed in Perinatal Medicine, Faculty of Medicine, Pontificia Universidad Javeriana, Hospital Universitario San Ignacio, Bogotá 110231, Colombia
[10] Department of Obstetrics and Gynecology, Faculty of Medicine, Pontificia Universidad Javeriana, Hospital Universitario San Ignacio, Bogotá 110231, Colombia
[11] Department of Pathology, Faculty of Medicine, Pontificia Universidad Javeriana, Hospital Universitario San Ignacio, Bogotá 110231, Colombia
[12] Department of Physiological Sciences, Faculty of Medicine, Pontificia Universidad Javeriana, Bogotá 110231, Colombia
* Correspondence: payala@javeriana.edu.co (A.-R.P.); dmennickent@ucsc.cl (M.D.)
† These authors contributed equally to this work.

Table S1. Variables considered as input or target features for machine learning modelling.

Variable	Used as Input	Used as Target
Maternal age	Yes	No
Marital status	Yes	No
Education level	Yes	No
Age at menarche	Yes	No
Sex of newborn	Yes	No
Occupation	Yes	No
BMI	Yes	No
Personal PE background	Yes	No
Family history of PE	Yes	No
Personal or family history of IUGR	Yes	No
Personal history of chronic hypertension	Yes	No
Personal history of allergy	Yes	No
Personal history of migraine	Yes	No
Personal history of hypothyroidism	Yes	No
Family history of cardiovascular disease	Yes	No
Family history of spontaneous abortion	Yes	No
Family history of obit or perinatal death	Yes	No
Family history of preterm birth	Yes	No
Personal and family history of diabetes	Yes	No
Family history of cancer	Yes	No
Family history of hypertension	Yes	No
Pre-pregnancy and first trimester cigarette exposure	Yes	No
Pre-pregnancy and first trimester alcohol consumption	Yes	No
Primigravidity	Yes	No
Primipaternity	Yes	No
Number of abortions	Yes	No
Number of pregnancies	Yes	No
Number of sexual partners	Yes	No
Socioeconomic status	Yes	No
Time in relationship with the infant’s father	Yes	No
Gestational age at delivery	No	Yes
Newborn weight	No	Yes
Vital status of the newborn	No	Yes
Type of delivery	No	Yes
Malformations of the newborn	No	Yes
IUGR	No	Yes
Eclampsia or HELLP	No	Yes
PE subtype (EOP or LOP)	No	Yes

BMI: body mass index. PE: preeclampsia. IUGR: intrauterine growth restriction. HELLP: hemolysis, elevated liver enzymes, low platelet counts syndrome. EOP: early-onset preeclampsia. LOP: late-onset preeclampsia.

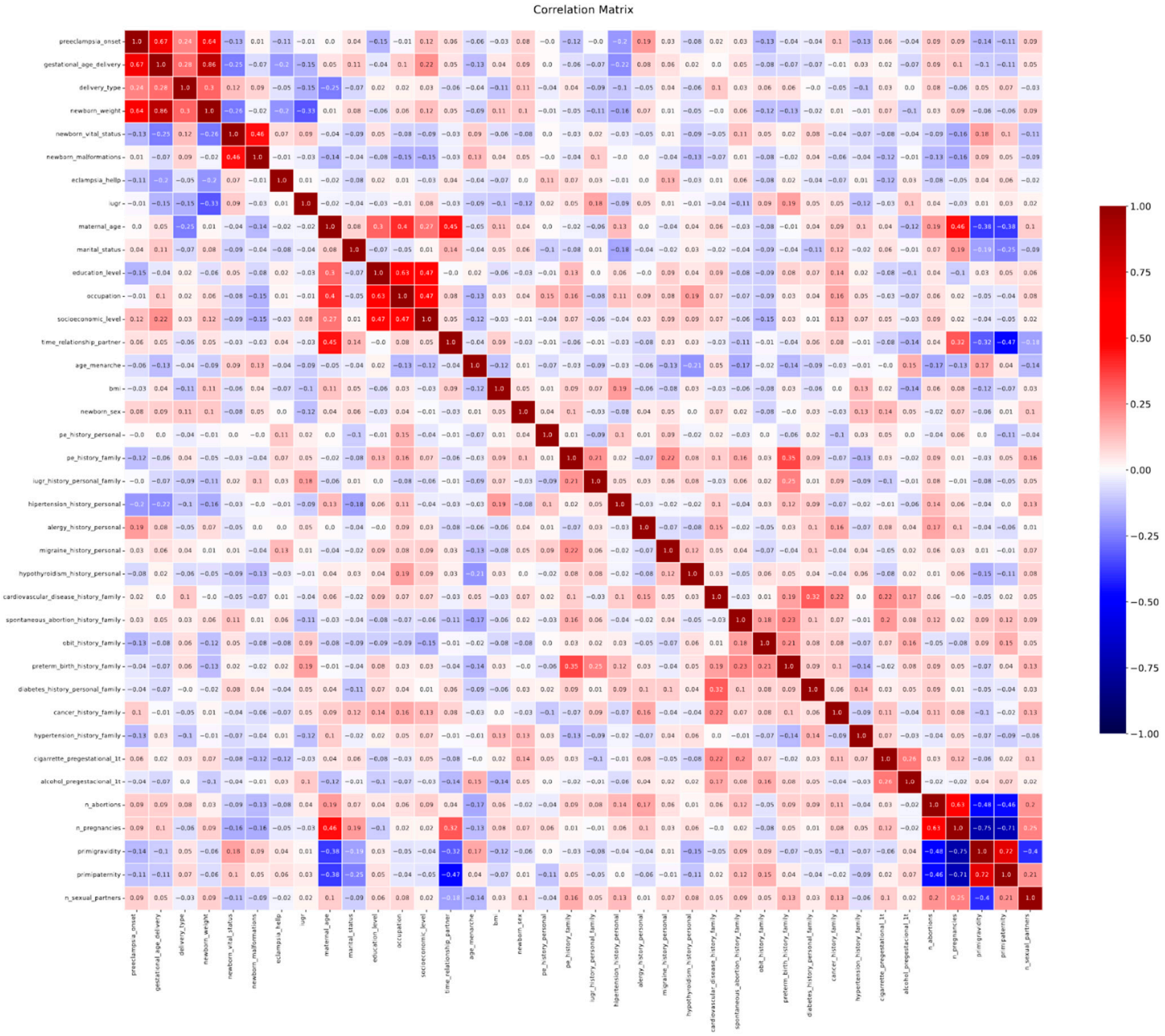

Figure S1. Correlation matrix between all risk factors and outcomes considered in this study.

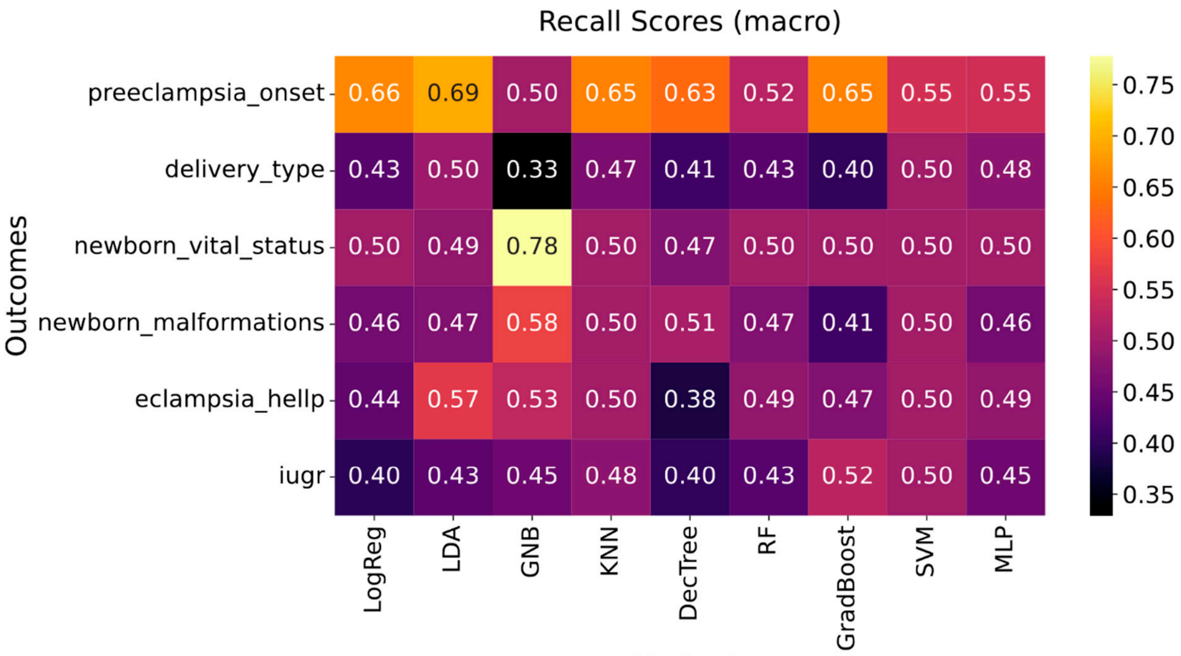

Figure S2. Recall of the machine learning models developed for the prediction of categorical outcomes, using all risk factors as predictors. *HELLP: hemolysis, elevated liver enzymes, low platelet counts syndrome. IUGR: intrauterine growth restriction. LogReg: logistic regression; LDA: linear discriminant analysis. GNB: Gaussian Naïve Bayes. KNN: k-nearest neighbors. DecTree: decision tree. RF: random forest. GradBosost: gradient boosting. SVM: support vector machine. MLP: simple multilayer perceptron.*

Table S2. Regression metrics of the machine learning models developed for the prediction of continuous outcomes, using all risk factors as predictors.

	**Regression Technique and Metrics**	
**Outcome**	**Random Forest Regressor**	**Gradient Boosting Regressor**
**Gestational age at delivery (weeks of gestation)**	MSE=15.97<br>RMSE=4.00<br>MAE=3.16<br>R2=0.05	MSE=20.10<br>RMSE=4.48<br>MAE=3.63<br>R2=-0.20
**Newborn's weight (g)**	MSE=697910.97<br>RMSE=835.41<br>MAE=705.93<br>R2=0.00	MSE=750873.97<br>RMSE=866.53<br>MAE=734.14<br>R2=-0.07

MSE: mean squared error; RMSE: root mean square error; MAE: mean absolute error; R2: coefficient of determination.

Table S3. Regression metrics of the machine learning models developed for the prediction of continuous outcomes, after variable selection.

	**Regression Technique and Metrics**	
**Outcome**	**Random Forest Regressor**	**Gradient Boosting Regressor**
**Gestational age at delivery (weeks of gestation)**	MSE=16.48<br>RMSE=4.06<br>MAE=3.27<br>R2=0.02	MSE=17.49<br>RMSE=4.18<br>MAE=3.10<br>R2=-0.04
**Newborn's weight (g)**	MSE=763013.95<br>RMSE=873.51<br>MAE=739.23<br>R2=-0.09	MSE=808143.05<br>RMSE=898.97<br>MAE=737.94<br>R2=-0.15

MSE: mean squared error; RMSE: root mean square error; MAE: mean absolute error; R2: coefficient of determination.
